# Predictors of treatment response following aspiration sclerotherapy of hepatic cysts: an international pooled analysis of individual patient data

**DOI:** 10.1007/s00330-016-4363-x

**Published:** 2016-05-14

**Authors:** Titus F. M. Wijnands, Maxime Ronot, Tom J. G. Gevers, Julie Benzimra, Leo J. Schultze Kool, Valérie Vilgrain, Joost P. H. Drenth

**Affiliations:** 1 Department of Gastroenterology and Hepatology, Radboud University Medical Center, P.O. Box 9101, code 455, 6500 HB Nijmegen, The Netherlands; 2 Department of Radiology, Beaujon University Hospitals Paris Nord Val de Seine, Clichy, France; 3 Department of Radiology, Radboud University Medical Center, Nijmegen, The Netherlands

**Keywords:** Hepatic cyst, Polycystic liver disease, Interventional, Drainage, Sclerotherapy

## Abstract

**Objectives:**

To identify predictive variables of treatment response following aspiration sclerotherapy of large symptomatic hepatic cysts.

**Methods:**

We collected individual patient data from two tertiary referral centres and included all patients treated with aspiration sclerotherapy of a large (>5 cm), symptomatic hepatic cyst. At six months, clinical response was defined as complete or incomplete. Secondary, suboptimal technical response was defined as lower quartile of cyst reduction. Predictive variables of clinical and technical response were analyzed by logistic regression analysis.

**Results:**

We included 86 patients (58 ± 10 years; female 90 %). Complete clinical response rate was 55 %. Median cyst diameter and volume reduction were 71 % (IQR 50-87 %) and 98 % (IRQ 88-100 %), respectively. Patients with complete clinical response had a significantly higher cyst reduction compared to incomplete responders (OR 1.02, 95 % CI 1.00-1.04). Aspiration of haemorrhagic cyst fluid (OR 4.39, 95 % CI 1.34-14.39) or a lower cyst reduction at one month (OR 1.06, 95 % CI 1.02-1.10) was associated with a suboptimal technical response at six months.

**Conclusion:**

Complete clinical response is associated with effective cyst reduction. Aspiration of haemorrhagic cyst fluid or a restricted diameter reduction at one month predicts a suboptimal technical treatment response, however, these variables did not predict symptom disappearance.

***Key Points*:**

• *Aspiration sclerotherapy of hepatic cysts shows excellent clinical and technical efficacy.*

• *Optimal clinical responders have a markedly higher cyst reduction.*

• *Haemorrhagic aspirate and a strong fluid reaccumulation predict suboptimal cyst reduction.*

**Electronic supplementary material:**

The online version of this article (doi:10.1007/s00330-016-4363-x) contains supplementary material, which is available to authorized users.

## Introduction

Hepatic cysts are fluid-filled cavities that arise from congenital malformations of biliary ducts [1]. The estimated prevalence of these benign, non-parasitic lesions is 2-18 % in the general population [2–4]. Hepatic cysts occur solitary or as multiple lesions in the context of polycystic liver disease (PLD) [5]. Aspiration sclerotherapy is a minimally invasive treatment option and serves as first-choice treatment for symptomatic hepatic cysts [6–8]. Aspiration sclerotherapy combines percutaneous drainage of cyst fluid with subsequent instillation of a sclerosing agent that destroys the inner cyst lining. This treatment is effective and safe [9–14]. There are two components that are important to the perceived success of aspiration sclerotherapy treatment. First, clinical response as experienced by the patient, and second, technical response in terms of reduction of the treated cyst. It is not well understood which factors determine clinical success or are important for technical response. Due to the infrequency of large symptomatic hepatic cysts, published studies are relatively small, which is an obstacle on the road to identification of predictive factors. In this study, we collected individual patient data from two observational cohort studies with comparable treatment protocols and follow-up measurements. The goal of this study was to identify variables that predict response of aspiration sclerotherapy of large symptomatic hepatic cysts.

## Materials and methods

### Patients and study design

We performed a pooled analysis of data collected from patients that consecutively underwent aspiration sclerotherapy in two tertiary referral centres: Radboud University Medical Center, Nijmegen, the Netherlands (centre 1) and University Hospitals Paris Nord Val de Seine, Beaujon, Clichy, France (centre 2) (Table 1). Centre 1 prospectively included patients treated within a timeframe of June 2012 to March 2014, centre 2 retrospectively included patients treated from December 2003 to September 2011. Within this timeframe the treatment protocol remained unchanged. For this analysis we included a cohort (centre 2) that was previously published to report efficacy of aspiration sclerotherapy [13]. In the current study, we extended our data by pooling this sample with a second prospective cohort (centre 1). By this, we created a large sample size that was needed to evaluate predictors of treatment response.

**Table 1:** Design characteristics of included studies

	Centre 1	Centre 2
Number of patients	29	57
Design	Prospective cohort	Retrospective cohort
Type treatment	Single session, ethanol	Single session, ethanol
Population	Solitary cysts and PLD	Solitary cysts and PLD
Inclusion criteria	>18 y; simple, non-parasitic cyst	>18 y; simple, non-parasitic cyst
Endpoints	Clinical and technical outcome, safety	Clinical and technical outcome, safety
Timepoints	Baseline, one, and six months	Baseline, one, six, and 12 months

Abbreviations: PLD, polycystic liver disease; y: years

**Fig. 1:**
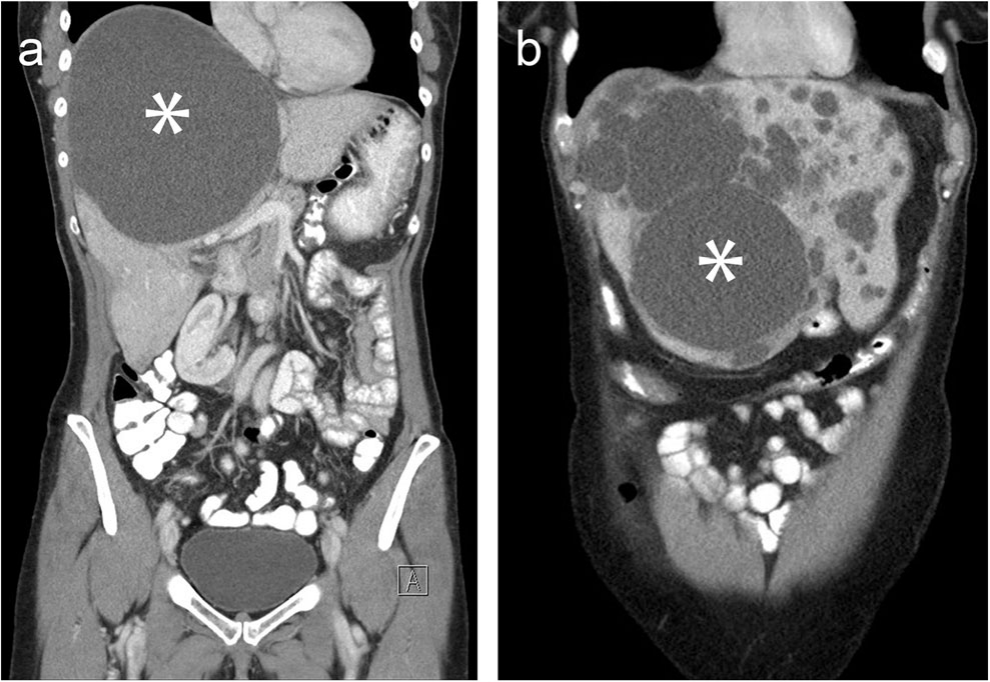
Computed tomography demonstrating (asterisk) a solitary (a) and dominant hepatic cyst in polycystic liver disease (b)

Included patients were aged over 18 years and underwent aspiration sclerotherapy of a simple, symptomatic, solitary or dominant hepatic cyst larger than 5 cm (Fig. 1). A simple cyst was diagnosed by ultrasound (US), computed tomography (CT), or magnetic resonance imaging (MRI) based on the following criteria: a well-circumscribed anechoic lesion with increased posterior echo enhancement and no evidence of mural nodularity on US; a water-density (−10 to 10 HU) lesion with sharply defined margins and smooth thin walls with no contrast enhancement or septations on CT; or a homogeneous lesion, hypointense on T1-weighted images and hyperintense on T2-weighted images without contrast enhancement or septations on MRI [15, 16]. A history of a previous cyst haemorrhage was not considered an exclusion criterion. The diagnosis of a haemorrhagic cyst was based on pre-defined criteria [16]. If deemed necessary, serology and/or more extensive imaging was applied to rule out hydatid or (pre-)malignant lesions. Parasitic or neoplastic cysts were not treated by aspiration sclerotherapy. In PLD patients (>20 cysts), the largest cyst was regarded as dominant cyst. A patient was excluded from analysis if the treated cyst could not be identified following treatment or was lost to follow-up. If a patient underwent two or more aspiration sclerotherapy procedures of the same cyst, only the first procedure was included in this analysis. The institutional review board of both participating centres approved this study and requirement for informed consent was waived.

### Aspiration sclerotherapy

**Fig. 2:**
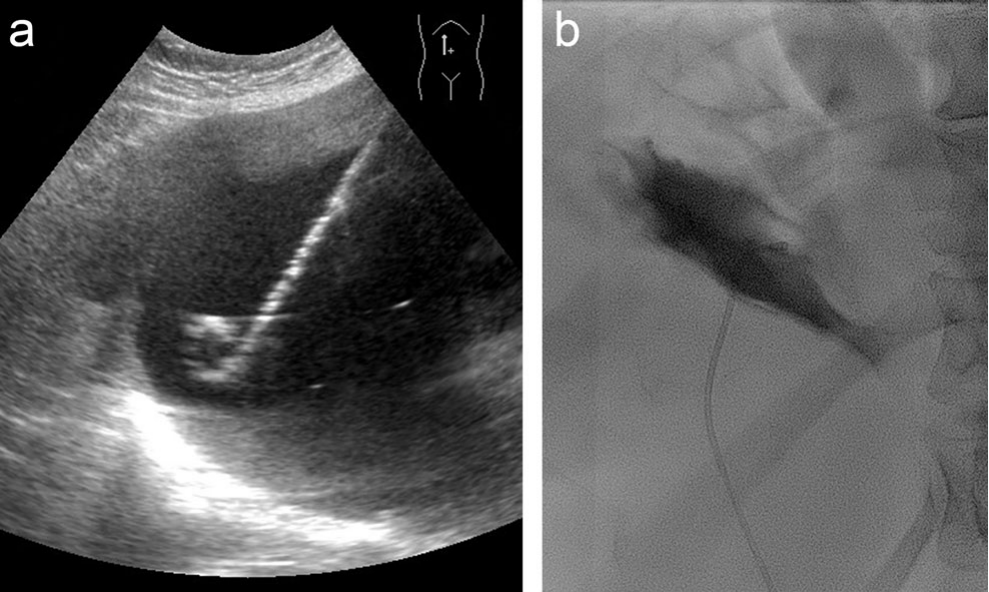
Ultrasonography-guided placement of pigtail catheter to perform aspiration (a) followed by fluoroscopy demonstrating a collapsed hepatic cyst without leakage (b)

Patients were informed about possible related risks and agreed to undergo aspiration sclerotherapy. In both centres, patients underwent aspiration sclerotherapy of a single hepatic cyst following a standardized single-session percutaneous ethanol sclerotherapy protocol [13]. The intervention was performed with local anaesthesia and conscious sedation in centre 1, and under general anaesthesia in centre 2. The cyst was localized by US and punctured with a 5-French pigtail catheter (centre 1: Cook Medical, Bloomington, United States of America; centre 2: Cordis Corporation, Bridgewater, United States of America) to perform complete fluid drainage of the hepatic cyst (Fig. 2). Aspirated fluid was classified as clear or haemorrhagic (red or brown) fluid and evaluated by microscopy. Subsequently, we performed fluoroscopy following instillation of contrast fluid (centre 1: Iomeron 300, Bracco Imaging, Konstanz, Germany; centre 2: Iobitridol, xenetix 350, Guerbet, France) to rule out leakage or communication with vasculature or the biliary tree (Fig. 2). Sclerotherapy was performed by injecting 96-100 % ethanol (centre 1: up to 50 mL, 10 minutes; centre 2: up to 150 mL, 60 minutes) in the cyst. Following sclerotherapy, all ethanol was re-aspirated and the catheter was removed from the patient. Patients were discharged one day following the procedure.

### Baseline and follow-up study visits

At baseline and at one and six months following aspiration sclerotherapy, the patient visited the outpatient clinic to evaluate clinical and technical parameters. In both centres, overall clinical response was assessed at six months by standardized instruments and defined as “disappearance”, “reduction”, “no change”, or “aggravation” of symptoms [13, 17]. If a patient had not completed the questionnaire, we retrospectively reviewed the patients’ clinical chart (*n* = 10). Detailed information of symptom assessment can be found in the supplemental material.

**Fig. 3:**
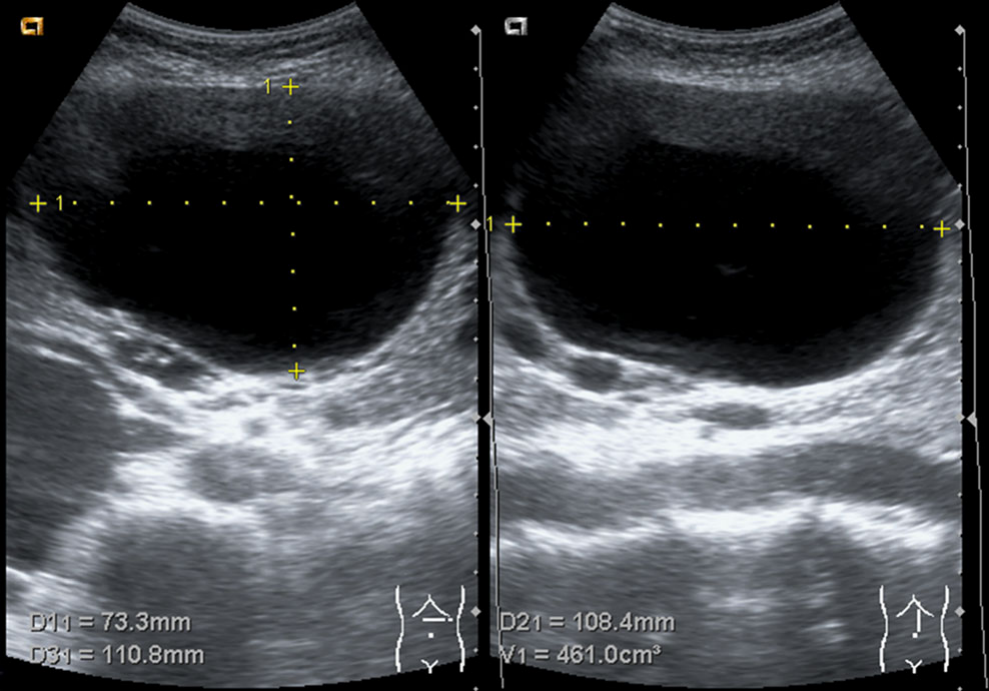
Hepatic cyst diameter measurement by ultrasonography; transversal (left) and coronal (right) plane to estimate hepatic cyst volume

The maximal cyst diameter of the treated cyst was measured during all visits (Fig. 3). Subsequently, we estimated cyst volume by multiplying available orthogonal diameters by 0.523 using the ellipsoid volume formula (d1*d2*d3*0.523). If an orthogonal diameter was missing, we substituted this value by the maximal diameter. In centre 1, all measurements were performed by US (Acuson X150™, Siemens Healthcare, Erlangen, Germany) by the same investigator. In centre 2, US (Aixplorer ultrasound system, SuperSonic Imagine, Aix-en-Provence, France; Aplio, Toshiba Medical system, Tokyo, Japan), CT (Advantage LightSpeed VCT, GE Healthcare, Milwaukee, United States of America), or MRI (1.5-T imager, interna, Philips Healthcare, Best, the Netherlands) were applied and measured by two investigators. Both centres monitored adverse events until six months following treatment.

### Outcome variables

The primary outcome was the overall clinical response at six months following treatment. To predict optimal clinical success, we divided clinical outcome into two groups: *complete clinical responders* that reported “disappearance” or *incomplete clinical responders* that reported “reduction”, “no change” or “aggravation” of symptoms.

As a secondary outcome, technical response was evaluated by the proportional cyst reduction at six months. From a technical point of view, we were mainly interested in patients with a low (suboptimal) outcome to identify factors that are associated with lower efficacy of the procedure. To do so, we grouped technical response into patients with or without a suboptimal response, defined by a cyst diameter reduction in the lower quartile of the total sample. Secondly, we repeated this evaluation with cyst volume reduction measurements.

### Predicting variables

The following variables were selected for analysis: age at treatment (years), gender, PLD (defined as > 20 cysts), previous drainage of the treated cyst, baseline cyst diameter (cm), location of the treated cyst (right or left liver lobe), haemorrhagic aspect of aspirated cyst fluid, volume of injected ethanol (mL), and proportional diameter reduction (%) at one and six month(s) following aspiration sclerotherapy.

### Data collection

Both centres completed an electronic form containing all variables of interest. A pooled dataset was created by the initiating investigator. If possible, data inconsistencies were corrected in agreement with both centres.

### Statistical analysis

Baseline characteristics are given in mean (± SD) or median (± IQR) according to distribution. Patients characterized as complete clinical responder were compared with patients with an incomplete clinical response on predefined predicting variables by univariate analysis. Similarly, we compared patients with or without a suboptimal technical response. Variables were selected as fixed effects in logistic regression analysis (*generalized linear mixed models*) if a *p*-value < 0.2 was found. To identify predictors of clinical and technical response, we used backward selection by excluding variables with a *p*-value > 0.05 in the multivariate analysis. The variable centre retained in univariate and multivariate analysis as a random effect to correct for possible differences between centres. Within groups of symptomatic relief, we compared proportional cyst diameter at six months by the non-parametric *Kruskal-Wallis* test. In addition, we explored whether a suboptimal technical response was associated with clinical response using the *Pearson’s chi-square test.* Finally, to confirm our findings, we repeated all uni- and multivariate analyses using cyst volume measurements instead of diameter measurements. Statistical analyses were performed using IBM SPSS software, version 20.0. *P*-values were two-tailed and a value < 0.05 was considered statistically significant.

## Results

### Baseline characteristics

**Fig. 4:**
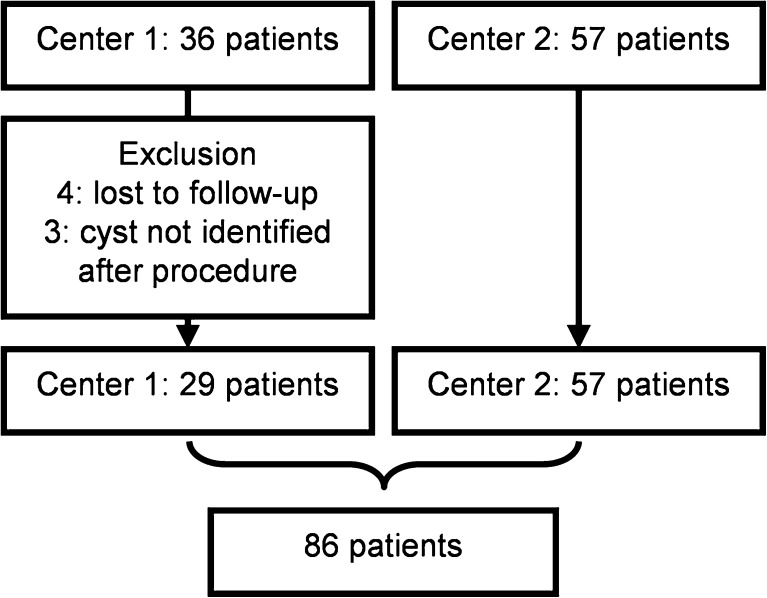
Overview of included patients

A total of 93 patients was retrieved from both centres. Seven patients were excluded from analysis (Fig. 4). Reasons for exclusion were loss to follow-up (*n* = 4) or inability to identify the treated cyst from other surrounding cysts following aspiration sclerotherapy (*n* = 3). This resulted in a pooled sample of 86 patients with a mean age at treatment of 57.8 (range 28 - 80) years (Table 2). Female patients (*n* = 77, 89.5 %) had a mean age of 57.0 (range 28 - 80) years, male patients (*n* = 9) had a mean age of 65.0 (57 – 79) years.

**Table 2:** Demographics (*n* = 86)

Age at treatment, mean (SD)	57.8 (10.2)
Female gender, *n* (%)	77 (89.5 %)
PLD, *n* (%)	33 (38.4 %)
Previous drainage treated cyst, *n* (%)	19 (22.1 %)
Location cyst in right liver lobe, *n* (%)	60 (69.8 %)
Cyst diameter baseline, median (IQR)	11.0 (9.3-15.0)
Cyst volume baseline, median (IRQ)	696 (381-1584)
Volume aspirate, median (IQR)	500 (300-1200)
Haemorrhagic aspect of cyst fluid, *n* (%)	32 (38.1 %)^1^
Volume of ethanol, median (IRQ)	70 (50-110)

^1^Cyst fluid aspect was not characterized in two patients. Age is presented in years, diameters in centimeters, and volumes in milliliters. Abbreviations: PLD, polycystic liver disease; SD, standard deviation; IQR, interquartile range

Cysts were most frequently localized in the right hepatic lobe (69.8 %). PLD was present in 38.4 %. The median maximal diameter at baseline of the treated hepatic cyst was 11.0 cm (9.3-15.0 cm) corresponding with a median estimated baseline volume of 696 mL (381-1584 mL). Aspect of cyst aspirate was assessed in 84 cases: 52 (61.9 %) aspirates were classified as clear and 32 (38.1 %) aspirates contained haemorrhagic constituents. This was confirmed by microscopic evaluation.

### Overall clinical and technical response

At six months, 47 patients (54.7 %) reported disappearance of symptoms and were defined as complete clinical responders. Thirty patients (34.9 %) reported reduction, and nine patients (10.4 %) had no change of symptoms following aspiration sclerotherapy and were collectively defined as incomplete clinical responders. In total, 89.6 % of patients had either disappearance or reduction of symptoms. No patients reported aggravation of symptoms.

Median cyst diameter decreased from 11.0 cm (9.3-15.0 cm) to 8.6 cm (6.0-11.0 cm) one month following aspiration sclerotherapy, corresponding to a median proportional reduction of 29.7 % (17.4-39.2 %). Cyst diameter regression continued to a median cyst diameter of 3.5 cm (1.0-6.4 cm) six months following aspiration sclerotherapy, corresponding to a proportional reduction of 70.7 % (50.0-87.2 %). In line, median proportional cyst volume regressed by 68.0 % (40.4-78.4 %) to 264 mL (103-605 mL) in the first month. At six months, median volume regressed to 22 mL (1-101 mL) corresponding with a proportional reduction of 97.8 % (87.5-99.8 %). Overall, centre 2 reported a higher cyst reduction at six months than centre 1: 99 % (94-100) vs 92 % (68-98 %), respectively.

### Predictors of complete clinical response

After univariate analysis, we selected location of the treated cyst, volume of injected ethanol, proportional diameter reduction at one month, and proportional diameter reduction at six months for subsequent regression analysis (Table 3). Multivariate analysis identified that an increased proportional diameter reduction at six months was associated with a complete clinical response (OR 1.02, 95 % CI 1.00-1.04, *p* = 0.046). When we replaced proportional diameter reduction at six months for volume measurements, statistical significance was lost (OR 1.02, 95 % CI 0.99-1.05, *p* = 0.665, Supplementary Table 1). We observed no significant centre effect (*p* = 0.13).

**Table 3:** Predictors of complete clinical response, univariate and multivariate analysis

Independent variable	Complete response (*n* = 47)	Incomplete response (*n* = 39)	Univariate analysis, *p-*value	Multivariate analysis, Odds ratio (95 % CI)	Multivariate analysis, *p-*value
Age at treatment, mean (SD)	57.9 (10.3)	57.7 (10.3)	0.962		
Female gender, *n* (%)	44 (93.6)	33 (84.6)	0.227		
PLD, *n* (%)	16 (34.0)	17 (43.6)	0.402		
Previous drainage of treated cyst, *n* (%)	9 (23.1)	10 (21.3)	0.889		
Baseline cyst diameter, mean (SD)	11.8 (3.5)	12.7 (4.0)	0.260		
Location cyst in right liver lobe, *n* (%)	30 (63.8)	30 (76.9)	0.179	1.74 (0.62-4.86)	0.288
Haemorrhagic aspect of cyst fluid, *n* (%)^1^	17 (63.0)	15 (60.5)	0.940		
Volume of ethanol, median (IQR)	80.0 (40.0-110.0)	50.0 (50.0-110.0)	0.143	0.99 (0.97-1.01)	0.319
Proportional diameter reduction one month, median (IQR)	32.1 (21.4-45.5)	27.6 (11.8-36.7)	0.118	1.01 (0.98-1.04)	0.425
Proportional diameter reduction six months, median (IQR)	78.6 (60.0-92.3)	59.1 (39.2-81.3)	0.046	1.02 (1.00-1.04)	**0.046**

^1^Cyst fluid aspect was not characterized in two patients. Age is presented in years, diameter in centimeters, volume in milliliters and proportional reductions in percentages. Abbreviations: PLD, polycystic liver disease; SD, standard deviation; IQR, interquartile range

**Fig. 5:**
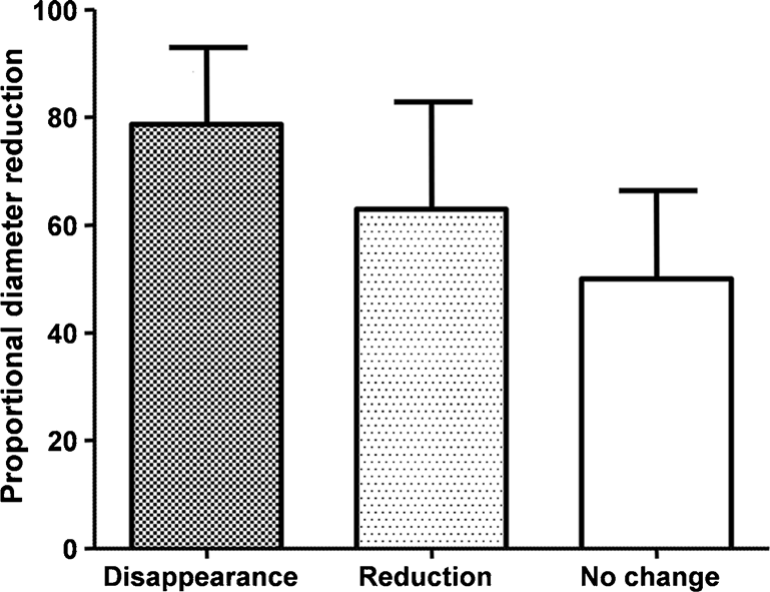
Proportional cyst diameter reduction within groups of symptom relief at six months

Further explorative analysis revealed that proportional diameter reduction at six months was 78.6 % (60.0-92.9 %) in patients with symptom disappearance, 62.9 % (43.4-82.8 %) in patients with symptom reduction, and 50 % (24.5-66.4 %) in patients who reported no change of symptoms (*p*-value = 0.01, Fig. 5). In accordance with our diametric analysis, patients with symptom disappearance showed a statistically significant higher median volume reduction at six months of 99.0 % (94.1-100 %), compared to 94.4 % (79.1-99.5 %) and 87.5 % (60.6-95.9 %) in patients with reduction or no change of symptoms, respectively (*p*-value = 0.02).

### Predictors of suboptimal technical response

The lower quartile in technical response (*n* = 22) was defined by a proportional diameter reduction of < 50.0 % and a volume reduction of < 87.5 %, characterized as suboptimal technical response.

Baseline cyst diameter, proportional cyst diameter reduction after one month, and aspect of cyst fluid were selected for subsequent regression analysis (Table 4). By multivariate analysis, we identified that patients with lower diameter reduction at one month had higher risk of having a suboptimal technical response at six months (OR 1.06, 95 % CI 1.02-1.10, *p* < 0.01). In addition, cysts containing haemorrhagic fluid were associated with a suboptimal technical response (OR 4.39, 95 % CI 1.34-14.39, *p* = 0.02). We confirmed our findings when diameter outcome and predictors were replaced by volume measurements (Supplementary Table 2). A suboptimal technical response was predicted by a lower volume reduction at one month (OR 1.04, 95 % CI 1.02-1.06, *p* < 0.01) and haemorrhagic cyst fluid (OR 4.78, 95 % CI 1.38-16.54, *p* = 0.01). Again, we found no significant centre effect (*p* = 0.06). Finally, explorative analysis revealed that 8 of 22 (36.4 %) patients with a suboptimal technical response had complete clinical response, compared to 39 of 64 (60.9 %) patients without a suboptimal technical response, a difference that was statistically significant (*p* = 0.046).

**Table 4:** Predictors of suboptimal technical response, univariate and multivariate analysis

Independent variable	Suboptimal response (*n* = 22)	No suboptimal response (*n* = 64)	Univariate analysis, *p-*value	Multivariate analysis, odds ratio (95 % CI)	Multivariate analysis, *p-*value
Age at treatment, mean (SD)	60.6 (9.8)	56.8 (10.2)	0.095	1.04 (0.98-1.10)	0.202
Female gender, *n* (%)	19 (86.4)	58 (90.6)	0.712		
PLD, *n* (%)	9 (40.9)	24 (34.5)	0.860		
Previous drainage of treated cyst, *n* (%)	4 (18.2)	15 (23.4)	0.536		
Baseline cyst diameter, mean (SD)	13.6 (4.4)	11.7 (3.4)	0.046	1.10 (0.94-1.29)	0.239
Location cyst in right liver lobe, *n* (%)	16 (72.7)	44 (68.8)	0.692		
Haemorrhagic aspect of cyst fluid, *n* (%)^1^	13 (59.1)	19 (29.7)	0.034	4.39 (1.34-14.39)	**0.015**
Volume of ethanol, median (IQR)	50.0 (47.5-110.0)	50.0 (50.0-110.0)	0.320		
Proportional diameter reduction one month, median (IQR)	17.1 (4.1-36.7)	32.0 (24.6-45.2)	0.009	1.06 (1.02-1.10)	**0.007**

^1^Cyst fluid aspect was not characterized in two patients. Age is presented in years, diameter in centimeters, volume in milliliters and proportional reductions in percentages. Abbreviations: PLD, polycystic liver disease; SD, standard deviation; IQR, interquartile range

### Safety

Overall, nine adverse events (10.7 %) occurred. Four patients developed an adverse event immediately after treatment, these included: hypotension (*n* = 2), transient fever (*n* = 1), and a cyst haemorrhage (*n* = 1). During follow-up, four patients reported pain between 2-24 days after treatment, in three of these patients a cyst haemorrhage was suspected based on clinical and radiological presentation. Finally, one patient developed an hepatic cyst infection (*n* = 1), which was treated adequately by antibiotic treatment. The remaining adverse events were managed by conservative treatment. All adverse events resolved without sequelae.

## Discussion

The primary finding of this study is that a strong hepatic cyst reduction following aspiration sclerotherapy predicts complete clinical response. We also identified that haemorrhagic cyst aspirate and a limited cyst size reduction at one month predicted suboptimal long-term cyst reduction but did not affect clinical response.

Clinical efficacy in this pooled cohort was high as 89.6 % of treated patients reported reduction of symptoms while symptoms disappeared completely in 54.7 %. The literature contains four large studies that assessed symptomatic response following aspiration sclerotherapy with various sclerosing agents. Similar to our results, these studies reported reduction of symptoms in 72.0-92.2 %, while 46.1-85.0 % had complete resolution of symptoms [7, 10, 18, 19]. However, these studies failed to investigate which factors contribute to clinical treatment response. We used the model of an individual patient data analysis with data coming from two independent cohorts, to demonstrate that a significant reduction of the treated cyst was associated to a better overall clinical response. Indeed, patients with a cyst diameter reduction over 50 % were more likely to have complete clinical response. This underlines that effective technical success is needed to increase the patients’ chance to benefit of treatment.

We observed a median proportional diameter reduction of 71 % at six months following aspiration sclerotherapy, corresponding with a volume reduction of 98 %. This strong reduction rate is comparable to other studies that evaluated long-term technical success of aspiration sclerotherapy. These studies achieved a proportional diameter reduction ranging from 54.8-85.7 % after 12-48 months [7, 20–22]. Correspondingly, studies that reported efficacy in terms of proportional volume reduction ranged from 90.8-98.9 % following 12-30 months [18, 23–25].

Sclerotherapy of hepatic cysts containing haemorrhagic fluid is not contraindicated and leads to reduction of the cyst [13]. Nonetheless, we found that drainage of haemorrhagic cyst fluid predicted an increased risk of a suboptimal technical treatment response. This may be the result of several factors. Due to increased viscosity, residues of haemorrhagic cyst fluid may remain in the cyst following drainage, leading to a diluted concentration of the sclerosing agent [7, 26]. Alternatively, clots or debris in the cyst may prevent contact with the cyst wall [10]. A spontaneous cyst bleeding is estimated to occur in around 8 % of hepatic cysts [27]. Remarkably, 32 (38 %) patients in our sample had cyst fluid containing haemorrhagic constituents. This relatively large proportion of haemorrhagic cyst fluid in treated patients may result of the symptomatic course following a cyst bleed, ultimately leading to treatment of the cyst. In this study, haemorrhagic content was not associated to a reduced clinical response. The present results validate the use of aspiration sclerotherapy in this subgroup of patients.

Furthermore, we found that a lower cyst reduction at one month predicted a suboptimal technical response at six months. Intracystic fluid re-accumulation directly following aspiration sclerotherapy is a well-known phenomenon that leads to a restricted cyst reduction in the first week(s) [28]. Although temporary, the cyst may relapse completely following aspiration sclerotherapy [24]. Previous studies speculated that secretory function of cyst-lining epithelial cells may persist following sclerotherapy; others considered fluid re-accumulation as a component of inflammatory response [7, 29]. Although transient in most cases, we found that a strong relapse predicted a suboptimal long-term cyst reduction.

Apart from aspiration sclerotherapy, surgical fenestration is often advocated as a primary treatment option for hepatic cysts. In a review of 43 studies (311 patients) evaluating efficacy and safety of cyst fenestration, reduction of symptoms was found in 92 % of treated patients [8]. This clinical response is consistent with our results. However, 22 % of laparoscopic procedures were converted to open fenestration and overall morbidity following cyst fenestration was reported in 23 % of cases, whereas in our study 11 % of patients experienced an adverse event. Our results suggest that aspiration sclerotherapy has comparable clinical efficacy with lower morbidity rates compared to its surgical alternative.

In this study, we confirm that aspiration sclerotherapy is highly effective in reducing symptoms. Therefore, we advocate that aspiration sclerotherapy should be primary treatment for patients with a symptomatic, solitary, or dominant hepatic cyst. In patients with haemorrhagic cyst fluid aspirate or a strong fluid reaccumulation following aspiration sclerotherapy, we advise increased attention. If symptoms recur, initiation of a subsequent aspiration sclerotherapy or referral for surgical treatment may be favourable.

The main strength of this study is that we pooled results of two similar studies from different countries and thereby increased the generalizability of our results. Moreover, this relatively large sample size enabled us to evaluate predictors of treatment response. Some limitations come with this study. Firstly, symptomatic change was evaluated by two different questionnaires. A detailed evaluation of specific symptoms could not be performed due to differences between these instruments. However, as both instruments assessed overall symptomatic response in a comparable standardized manner, we were able to pool results of overall clinical response and believe that possible introduced bias is limited. Secondly, due to small numbers of patients without reduction of symptoms, we grouped patients with “reduction” of symptoms and “no change” as *incomplete clinical responder*. As a result, we were unable to evaluate predicting variables of clinical non-responders. Furthermore, our follow-up extends over a period of six months and should ideally be validated over a longer period. However, we regard six months as sufficient as the most prominent cyst regression occurs within these first months and sustains thereafter [24]. Moreover, cyst diameter measurements were performed by different imaging modalities and investigators between centres; this could have led to differences in outcome measurements. Finally, duration of ethanol instillation differed between centre 1 and centre 2. As this was completely co-linear to centre, we could not include this variable in our analysis. A prolonged exposure of the sclerosing agent to the cyst wall may improve efficacy of treatment. On the other hand, previous studies stated that ethanol eliminates epithelial cells within minutes [24, 30]. The impact of duration of sclerotherapy on treatment efficacy remains to be tested.

To conclude, aspiration sclerotherapy of hepatic cysts effectively reduces symptoms in the vast majority of patients. Chance of complete clinical response is increased in patients with higher proportional cyst reduction. We identified that a limited cyst reduction in the first month and the presence of haemorrhagic cyst fluid predict a suboptimal technical treatment response, however, these variables did not predict symptom disappearance.

## Electronic supplementary material

Below is the link to the electronic supplementary material.

ESM 1 (DOCX 20 kb)

## Abbreviations

CT: Computed tomography
MRI: Magnetic resonance imaging
PLD: Polycystic liver disease
US: Ultrasound
