# Supplementary material for: Predictors of treatment response following aspiration sclerotherapy of hepatic cysts: an international pooled analysis of individual patient data
Source: Eur Radiol. 2016 May 14;27(2):741–8. doi: 10.1007/s00330-016-4363-x (PMC5209423; doi:10.1007/s00330-016-4363-x)
Supplement: Supplementary file 1 — (DOCX 20 kb) [file 330_2016_4363_MOESM1_ESM.docx]

**Predictors of treatment response following aspiration sclerotherapy of hepatic cysts: an international pooled analysis of individual patient data**

**SUPPLEMENTAL MATERIAL**

**Clinical response evaluation**

In center 1, the gastrointestinal symptoms (GIS) questionnaire was applied evaluating 11 abdominal symptoms on an adjectival scale ranging from 0 (“none”) to 6 (“severe”) [1]. One question was added evaluating overall clinical response of symptoms (“decrease”, “no change” or “aggravation” of symptoms) following AS. Likewise, in center 2, symptomatic change (“disappearance”, “persistence but decrease”, “no change” or “aggravation” of symptoms) following AS was assessed [2].

To pool results of overall symptomatic response, we re-evaluated the GIS scores from patients in center 1 who had characterized their symptoms as “decrease” and determined if a patient had “disappearance” or “persistence but decrease” of symptoms. If a patient had not completed the GIS questionnaire, we retrospectively assessed symptomatic relief by reviewing the patient’s chart.

| **Supplementary** **Table 1** Predictors of complete clinical response, confirmatory univariate and multivariate analysis using cyst volume variables | | | | | |
| --- | --- | --- | --- | --- | --- |
| Independent variable | Complete response  (*n* = 47) | Incomplete response (*n* = 39) | Univariate analysis, *p-*value | Multivariate analysis, odds ratio (95% CI) | Multivariate analysis, *p-*value |
| Age at treatment, mean (SD) | 57.9 (10.3) | 57.7 (10.3) | 0.962 |  |  |
| Female gender, *n* (%) | 44 (93.6) | 33 (84.6) | 0.227 |  |  |
| PLD, *n* (%) | 16 (34.0) | 17 (43.6) | 0.402 |  |  |
| Previous drainage of treated cyst, *n* (%) | 9 (23.1) | 10 (21.3) | 0.889 |  |  |
| Baseline cyst volume, median (IQR) | 696 (381-1435) | 696 (407-1765) | 0.309 |  |  |
| Location cyst in right liver lobe, *n* (%) | 30 (63.8) | 30 (76.9) | 0.179 | 1.89 (0.67-5.29) | 0.224 |
| Hemorrhagic aspect of cyst fluid, *n* (%)^1^ | 17 (63.0) | 15 (60.5) | 0.940 |  |  |
| Volume of ethanol, median (IQR) | 80.0 (40.0-110.0) | 50.0 (50.0-110.0) | 0.143 | 0.99 (0.98-1.01) | 0.270 |
| Proportional volume reduction one month, median (IQR) | 68.8 (51.5-83.8) | 63.6 (20.2-77.3) | 0.073 | 1.01 (0.99-1.03) | 0.189 |
| Proportional volume reduction six months, median (IQR) | 99.0 (94.1-100) | 93.2 (78.6-99.3) | 0.209 | 1.02 (0.99-1.05) | 0.665 |
| ^1^ Cyst fluid aspect was not characterized in two patients. Age is presented in years, diameter in centimeters, volume in milliliters and proportional reductions in percentages. Abbreviations: PLD, polycystic liver disease; SD, standard deviation; IQR, interquartile range | | | | | |

| **Supplementary Table 2** Predictors of suboptimal technical response, confirmatory univariate and multivariate analysis using cyst volume variables | | | | | |
| --- | --- | --- | --- | --- | --- |
| Independent variable | Suboptimal response (*n* = 22) | No suboptimal response (*n* = 64) | Univariate analysis, *p-*value | Multivariate analysis,  odds ratio (95% CI) | Multivariate analysis, *p-*value |
| Age at treatment, mean, (SD) | 60.6 (9.8) | 56.8 (10.2) | 0.095 |  |  |
| Female gender, *n* (%) | 19 (86.4) | 58 (90.6%) | 0.712 |  |  |
| PLD, *n* (%) | 9 (40.9) | 24 (37.5) | 0.860 |  |  |
| Previous drainage of treated cyst, *n* (%) | 4 (18.2) | 15 (23.4) | 0.536 |  |  |
| Baseline cyst volume, median (IQR) | 900 (467-2349) | 696 (381-1435) | 0.092 | 1.00 (1.00-1.00) | 0.307 |
| Location cyst in right liver lobe, *n* (%) | 16 (72.7) | 44 (68.8) | 0.692 |  |  |
| Hemorrhagic aspect of cyst fluid, *n* (%)^1^ | 13 (59.1) | 19 (30.6) | 0.034 | 4.78 (1.38-16.54) | **0.014** |
| Volume of ethanol, median (IQR) | 50.0 (47.5-110.0) | 90.0 (50.0-110.0) | 0.320 |  |  |
| Proportional volume reduction one month, median (IQR) | 40.4 (17.4-66.5) | 70.4 (58.2-83.5) | 0.002 | 1.04 (1.02-1.06) | **0.001** |
| ^1^ Cyst fluid aspect was not characterized in two patients. Age is presented in years, diameter in centimeters, volume in milliliters and proportional reductions in percentages. Abbreviations: PLD, polycystic liver disease; SD, standard deviation; IQR, interquartile range | | | | | |

**REFERENCES**

1. Bovenschen HJ, Janssen MJ, van Oijen MG, Laheij RJ, van Rossum LG, Jansen JB. Evaluation of a gastrointestinal symptoms questionnaire. Digestive diseases and sciences. 2006;51(9):1509-15.

2. Benzimra J, Ronot M, Fuks D, Abdel-Rehim M, Sibert A, Farges O, et al. Hepatic cysts treated with percutaneous ethanol sclerotherapy: time to extend the indications to haemorrhagic cysts and polycystic liver disease. Eur Radiol. 2014;24(5):1030-8.
